# The Quit Benefits Model: a Markov model for assessing the health benefits and health care cost savings of quitting smoking

**DOI:** 10.1186/1478-7547-5-2

**Published:** 2007-01-23

**Authors:** Susan F Hurley, Jane P Matthews

**Affiliations:** 1 Bainbridge Consultants, 532 Brunswick St, Fitzroy North, Victoria, 3068, Australia; 2 School of Population Health, The University of Melbourne, Parkville, Victoria, 3052, Australia

## Abstract

**Background:**

In response to the lack of comprehensive information about the health and economic benefits of quitting smoking for Australians, we developed the Quit Benefits Model (QBM).

**Methods:**

The QBM is a Markov model, programmed in TreeAge, that assesses the consequences of quitting in terms of cases avoided of the four most common smoking-associated diseases, deaths avoided, and quality-adjusted life-years (QALYs) and health care costs saved (in Australian dollars, A$). Quitting outcomes can be assessed for males and females in 14 five year age-groups from 15–19 to 80–84 years.

Exponential models, based on data from large case-control and cohort studies, were developed to estimate the decline over time after quitting in the risk of acute myocardial infarction (AMI), stroke, lung cancer, chronic obstructive pulmonary disease (COPD), and death. Australian data for the year 2001 were sourced for disease incidence and mortality and health care costs. Utility of life estimates were sourced from an international registry and a meta analysis.

In this paper, outcomes are reported for simulated subjects followed up for ten years after quitting smoking. Life-years, QALYs and costs were estimated with 0%, 3% and 5% per annum discount rates. Summary results are presented for a group of 1,000 simulated quitters chosen at random from the Australian population of smokers aged between 15 and 74.

**Results:**

For every 1,000 males chosen at random from the reference population who quit smoking, there is a an average saving in the first ten years following quitting of A$408,000 in health care costs associated with AMI, COPD, lung cancer and stroke, and a corresponding saving of A$328,000 for every 1,000 female quitters. The average saving per 1,000 random quitters is A$373,000. Overall 40 of these quitters will be spared a diagnosis of AMI, COPD, lung cancer and stroke in the first ten years following quitting, with an estimated saving of 47 life-years and 75 QALYs. Sensitivity analyses indicated that QBM predictions were robust to variations of ± 10% in parameter estimates.

**Conclusion:**

The QBM can answer many of the questions posed by Australian policy-makers and health program funders about the benefits of quitting, and is a useful tool to evaluate tobacco control programs. It can easily be re-programmed with updated information or a set of epidemiologic data from another country.

## Background

The prevalence of cigarette smoking in Australia has decreased markedly over the last two decades, from 35% in 1980 to 23% in 2001[1]. Policy-makers now often question the cost-effectiveness of proposed tobacco control activities, as well as the expected impact of current smoking patterns on specific diseases, health care utilisation and health care costs. A number of recent analyses have improved understanding in Australia of the potential benefits of further reductions in smoking rates. In 2003, Scollo outlined the economic and public policy rationale for greater investment by Australian governments in tobacco control. [2]. Hurley and colleagues,[3] in 2004, modelled the potential impact of reductions in smoking prevalence on Pharmaceutical Benefits Scheme (PBS) subsidies. They predicted that a 5% reduction in Australian smoking rates would reduce PBS spending on drugs for smoking-related cardiovascular disease by $4.5 billion over the following 40 years, a 17% reduction. Subsequently, Hurley also modelled the impact of a 5% reduction in Australian smoking rates on myocardial infarction and stroke hospitalisations and costs [4]. Predicted cost savings within 7 years were over $60 million.

Although these analyses interested policy-makers, they did not provide comprehensive quantitative predictions of the disease-specific health and health economic benefits of quitting smoking in Australia. Nor could we find a published model that could be adapted to provide such analyses. For example, the Tobacco Policy Model, developed by Tengs and colleagues for the United States population, predicts savings in smoking-associated mortality, morbidity and costs [5]. However, the impact of quitting on individual diseases is not modelled. Orme et al. developed the HECOS (health and economic consequences of smoking) model and used data from the United Kingdom to illustrate its capacity to evaluate smoking cessation strategies [6]. These researchers intended that the HECOS model would be widely accessible and modifiable for application to other countries, but the version we sourced could not be adapted to provide the analyses we needed. Johansson and colleagues developed a similar model to predict the health and economic consequences of smoking cessation in Sweden, [7] but it is not available for adaptation to other settings.

We therefore developed the Quit Benefits Model (QBM), which we conceptualised primarily as a tool to evaluate tobacco control programs where estimates of the number of quitters are available. We developed the QBM with similar rigour to that required for submissions to the Australian federal government for subsidisation of medicines,. [8] so that policy-makers could compare the health economic profile of a prospective tobacco control program with that of other health programs seeking government funding. The QBM can also be adapted to evaluate smoking cessation programs in other countries, if local epidemiologic data are available. The goals of this paper are therefore, first, to describe in detail the methodology used to develop the QBM, with particular emphasis on the sources of parameter estimates. Second, we present summaries of the benefits of quitting for Australian smokers. These summaries include health care cost savings per 1000 quitters and the number of cases of four smoking-associated diseases, deaths, life-years, and QALYs saved per 1000 quitters

## Methods

### Design of the QBM

The QBM assesses the effect of quitting on an individual smoker. The impacts on other people of a smoker quitting, through decreased exposure to environmental smoke or decreased in-utero exposure of an infant, are ignored. The following *outcomes *are assessed separately for male and female smokers and quitters, for various ages of quitting smoking, with different durations of follow-up and different discount rates for future benefits:

• Incidence of four diseases: acute myocardial infarction (AMI), stroke, lung cancer, and chronic obstructive pulmonary disease (COPD). These are the four most common causes of both death and morbidity attributable to smoking in Australia. In 1996, they accounted for almost 84% of the 16,875 deaths caused by smoking, and almost 78% of the 242,138 disability-adjusted life-years (DALYs) that were lost due to smoking [9].

• Total deaths, including deaths attributable to all smoking-related diseases, and deaths due to the above four diseases.

• Life expectancy.

• Quality-adjusted life-expectancy (QALE). Quality adjustments to life-expectancy were made based on the reduced utility of life associated with each of the four specified smoking-related diseases. A utility is formally defined as the quantitative measure of the strength of a person's preference for an outcome [10]. The utility of life is assumed to vary between 1 (for perfect health) and 0 (for death). The QBM assumes that the utilities of life for a healthy smoker and a healthy quitter are 1. Alternative approaches, such as using mean population age-group and sex specific utilities, or modeling possible temporary reductions in utility of life associated with quitting could be incorporated in future QBM analyses if suitable data were available. The QBM assumes that each of the four smoking-associated diseases has a utility less than 1, i.e. patients prefer perfect health to the disease.

• Direct health care costs associated with the above four diseases. Indirect costs due to time lost from work are not considered.

The consequences of quitting in terms of cases of the four specified diseases and deaths avoided, and life-years (LYs), quality-adjusted life-years (QALYs) and health care costs saved are calculated by subtracting the expected value of the outcome for a smoker from the expected value of the outcome for a quitter who ceases smoking at a given age and does not commence smoking again after that age.

**Figure 1:**
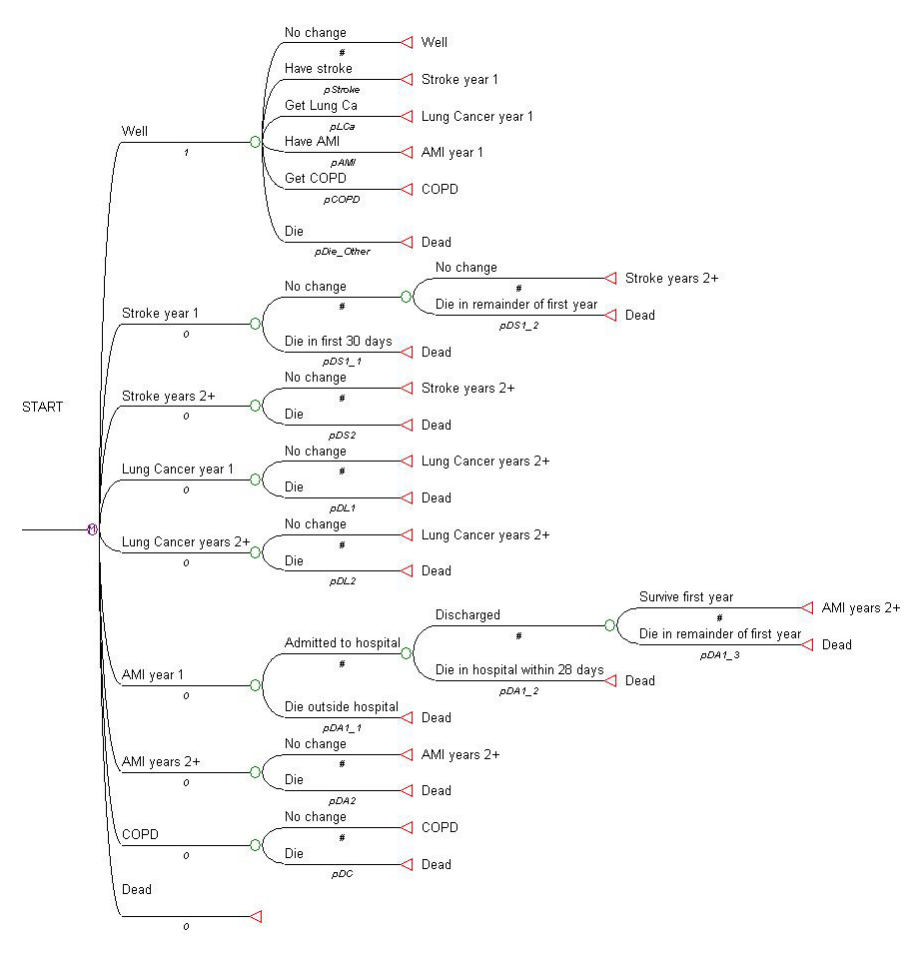
TreeAge Markov cycle tree for the QBM.

The QBM is a Markov-cycle tree model. Figure 1 shows the cycle tree for the QBM from TreeAge Pro, the software program that was used to analyse the model [11]. At the beginning of the analysis period, all subjects (smokers and quitters) are assumed to be in the health state "Well". During each subsequent one year period (or cycle) simulated subjects can either stay in the health state "Well", or, if one of the four smoking-related illnesses is diagnosed they move (transition) to the corresponding health state "Stroke year 1", "Lung Cancer year 1", "AMI year 1", or "COPD". If a subject dies, they move to the health state "Dead". Subjects who develop COPD are assumed to either stay in that state or die during subsequent cycles. The model is more complex for the other three smoking-associated diseases to reflect the fact that either the probabilities of death, or costs, or both, varied between or within subsequent cycles. Briefly, for subjects who develop lung cancer, the probability of death in a given year following diagnosis is dependent on the number of years post diagnosis. This was handled in TreeAge Pro by setting up a "tunnel variable" which counts the number of years survived since diagnosis and is used as the index in a look-up table for the probabilities of transitioning from lung cancer to death. For stroke, the first 30 days after diagnosis are associated with a higher probability of death than the remainder of the first year, and the subsequent annual probabilities of death depend on the number of years since the stroke occurred. Again this was managed in TreeAge Pro by setting up a "tunnel variable" to count the number of years since the stroke occurred. For subjects who have an AMI, the periods before admission to hospital (assumed to be the first 24 hours) and the first 28 days in hospital are associated with higher probabilities of death than subsequent periods. Beyond the first year after an AMI occurs, the annual probability of death does not vary with time since AMI. The data that underpinned the model design are described in more detail in the following sections of the Methods.

The QBM is analysed separately for smokers and quitters to calculate the expected value for each of the outcomes mentioned above, for males and females, for any of 14 five-year age-groups from 15–19 years to 80–84 years. The midpoint of each age-interval is used to estimate expected values for outcomes for each five-year age-group. A half-cycle correction is applied to allow for the fact that transitions between health states can take place at any time during the modeled one-year intervals [12]. Project-specific macros were developed in Excel to generate the various outcomes from the QBM using the TreeAge Pro Interactive Software. The results are summarized in Excel spreadsheets.

The QBM assumes that, for quitters, the risk of the four specified smoking-related diseases relative to smokers declines over time. We assume that following a diagnosis of lung cancer, stroke, AMI or COPD the probability of death due to the disease is independent of smoking or quitting status, but the risk of death from other causes declines over time for quitters relative to smokers. However, the type of data available to model death probabilities following a diagnosis of lung cancer or stroke differed from that available for AMI or COPD. The approach we took is described in detail under ***Probabilities of death for people with smoking-related diseases***. The QBM also assumes that the risk of death from causes other than the four specified smoking-related diseases is greater for smokers than for the general population, and that, for quitters, this excess risk declines over time. Health care costs and health state utilities for the four specified smoking-related diseases are assumed to be the same for smokers and quitters.

With the QBM it is possible to undertake a *time since quitting *analysis, where the expected value of each outcome is calculated for defined periods of time since quitting, or an analysis in which the expected value of each outcome is calculated up until subjects die or reach 85 years. Note that, even with the *time since quitting *analysis, in the QBM, no subjects are followed beyond the age of 85 years. In this paper, results are provided for a *time since quitting *analysis with a ten year follow-up. The 10-year follow-up period is long enough to show the beneficial impacts of quitting, but is hopefully short enough to remain within the time frame of policy-makers. Discount rates of 0%, 3% and 5% per annum were applied to life-years, QALYs and costs. Cases of disease and deaths avoided were estimated using Monte Carlo simulations and were not discounted. Results were averaged over 10,000 simulations.

### Model parameters

The most recent year for which key data were available (for example, lung cancer incidence and hospitalisation counts) was 2001, so it was used as the base year. The rates in 2001 were assumed to apply to all future years, i.e. possible future temporal trends in disease incidence and mortality were not incorporated in the model. The sources of parameter estimates are described below, and estimates for 50–54 year old males and females, for variables used to calculate cases of disease, deaths and life-expectancy are presented in Table 1.

**Table 1:** Model parameters for 50–54 year olds for estimation of cases of disease, deaths, and life-years

**Parameter**		**Males**	**Females**
Definition	Name		

**DISEASE-SPECIFIC**			

**AMI**			
Pr(AMI in next year for a smoker)	*pAMI*	0.0043	0.0012
Pr(AMI in next year for a quitter)*	*pAMI*	0.0015	0.0004
Pr(Die from an AMI before hospitalisation|have first AMI)	*pDA1_1*	0.1943	0.1744
Pr(Die in hospital from first AMI in first 28 days|have first AMI and hospitalised)	*pDA1_2*	0.0347	0.0335
Pr(Die from AMI in remainder of first year|discharged from hospital)	*pDA1_3*	0.0235	0.0149
Pr(Die from AMI in second year|survive first year)	*pDA2*	0.0235	0.0149
			
**Stroke**			
Pr(Stroke in next year for a smoker)	*pStroke*	0.0026	0.0016
Pr(Stroke in next year for a quitter)*	*pStroke*	0.0014	0.0008
Pr(Die from a stroke in first 30 days|have first stroke)	*pDS1_1*	0.2250	0.2250
Pr(Die from stroke in first year|survive first 30 days)	*pDS1_2*	0.1818	0.1818
Pr(Die from stroke in second year|survive first year)†	*pDS2*	0.0833	0.0833
			
**Lung cancer**			
Pr(Lung cancer in next year for a smoker)	*pLCa*	0.0016	0.0011
Pr(Lung cancer in next year for a quitter)	*pLCa*	0.6998	0.0006
Pr(Die from lung cancer in first year|have lung cancer	*pDL1*	0.5930	0.5099
Pr(Die from lung cancer in second year|survive first year)†	*pDL2*	0.4069	0.3602
			
**COPD**			
Pr(COPD in next year for a smoker)	*pCOPD*	0.0075	0.0062
Pr(COPD in next year for a quitter)	*pCOPD*	0.0052	0.0035
Pr(Die from COPD in year|have COPD)	*pDC*	0.0081	0.0060
			
**MORTALITY FROM OTHER CAUSES**			
Pr(Die from other causes in next year for a smoker)	*pDie_other*	0.0040	0.0029
Pr(Die from other causes in next year for a quitter)	*pDie_other*	0.0026	0.0024

Note that Probabilities vary once smokers or quitters age into the next stratum

Pr = probability

| = conditional on

* 5 years after quitting at age 45–49 years

+ Different probabilities for subsequent years

#### Probabilities of smoking-related diseases

Annual age-group and sex- specific incidence probabilities for the four specified smoking-related diseases were estimated for smokers and quitters. Probabilities were initially obtained for the general Australian population (i.e. for smokers, ex-smokers and never-smokers combined). Probabilities for smokers were then estimated by adjusting the population probabilities on the basis of smoking prevalence data and disease relative risks. Probabilities for quitters were estimated by adjusting the smokers' probabilities according to functions that describe the decline in risk of disease over time since quitting, relative to smokers.

##### AMI

The probabilities of first-ever AMI were estimated as the sum of the probabilities of first-ever fatal AMI (i.e. a fatal AMI that had not been preceded by a non-fatal AMI) and first-ever non-fatal AMI. The number of first-ever fatal AMIs was estimated by adjusting national ischaemic heart disease (IHD) 2001 mortality data (ICD 10 codes I20–I25) [13] for miscoding [14] and for previous AMIs [15]. To estimate the number of first non-fatal AMIs we first sourced data from the Australian Institute of Health and Welfare (AIHW) National Hospital Morbidity Database[16] on the number of hospital admissions for AMI in 2001–2 where the patient had a length of stay greater than two days and was discharged alive, as Jamrozik et al. [14] suggest that by excluding very short hospitalisations, patients re-admitted for revascularisation procedures are likely to be excluded. These data were then adjusted for miscoding of hospitalisations to AMI [14] and for previous AMIs [15].

##### Stroke

Probabilities of first-ever stroke (of any type) were obtained from NEMESIS, a population-based prospective study of the incidence of stroke in a region of Melbourne in 1996–97 [17]. This is the most recent population-based study of stroke incidence in Australia. The only other Australian study was conducted in Perth in 1989–90 [18]. The NEMESIS study provided similar incidence estimates, standardized for population age and sex distribution, to recent studies in Italy, Germany and Greece [17]. The NEMESIS study probabilities were assumed to apply to the year 2001, as the hospitalisation rate for stroke varied by < 1% over the subsequent years, 1997–98 to 2001–2002[16].

##### Lung cancer

Australian incidence probabilities for lung cancer for 2001 were sourced from the National Cancer Statistics Clearing House [19].

##### COPD

No population-based studies of the incidence of COPD in Australia have been conducted, and there are no routine data collections, such as exist for AMI, from which incidence could be estimated. Estimates of COPD incidence in 2003 for Australia, by age-group and sex were provided by Dr Theo Vos (University of Queensland, personal communication 24/8/2005). These estimates had been derived for the Australian 2003 Burden of Disease study (in progress). Vos and colleagues had originally estimated COPD prevalence for the 1994 Victorian Burden of Disease study, from population-based respiratory function data obtained from the Busselton health surveys. COPD was defined as forced expiratory volume in one second (FEV_1_) less than 70% of predicted, excluding those with a doctor's diagnosis of asthma. They then used DisMod (the precursor of DisMod II) to estimate COPD incidence in 1994. DisMod II is a software package developed by the World Health Organisation and its collaborators for the Global Burden of Disease 2000 study [20]. It is based on a set of differential equations, and enables users to calculate the complete epidemiology of a disease of interest given a minimum of three input variables. Vos and colleagues then estimated incidence in 2003 based on the observed changes in COPD mortality between 1994 and 2003.

We used log-linear interpolation between 1994 and 2003 to estimate the 2001 incidence for each age-and-sex-group.

#### Disease risks for smokers

The above estimates represented the annual incidence probability for the four specified smoking-related diseases, *D*_*P*, _for the Australian population. For AMI and stroke, the probability of disease (in a year) for smokers, *D*_*S*_, was estimated by first estimating the probability of disease for a never-smoker, *D*_*NS*_, according to the following formula:

*D*_*NS *_= *D*_*P*_/((1-*p*_*EX *_- *p*_*S*_) + *p*_EX_*RR_EX _+ *p*_S_*RR_S_)

Where *p*_*S *_is the prevalence of smokers, *p*_*EX *_is the prevalence of ex-smokers and RR_S _and RR_EX _are the risks of disease for smokers and ex-smokers, respectively, relative to never-smokers. Then:

*D*_*S *_= *D*_NS_* RR_S_

Smoking prevalence data were sourced from the Australian Bureau of Statistics 2001 National Health Survey (NHS) [21]. Dr Mohammad Siahpush (Cancer Council Victoria) queried the Remote Access Data Laboratory (RADL) using SPSS to obtain counts of smokers and ex-smokers by age-group and sex. The Australian Estimated Resident Population for 2001 was used to calculate proportions [22]. The relative risks of AMI and stroke were sourced from the meta analyses by English et al. [23].

The above method for estimating AMI and stroke disease risks for smokers was modified for lung cancer and COPD. Because of the long lead times from exposure (smoking) to development of lung cancer and COPD, substitution of the current smoking prevalence in the above equations to estimate *D*_*S *_for these diseases would be inappropriate, and in Australia, where smoking prevalence has decreased over time, would lead to underestimation of the proportions of these diseases attributable to smoking. Peto et al. [24] developed a method for indirect estimation of mortality from tobacco using published vital statistics, and part of this method was used by Dr Chris Stevenson from the AIHW to estimate Australian age-group and sex specific "synthetic prevalence" in 2001 for smoking. The "synthetic prevalence", *p*_*e*,_can be interpreted as the historical smoking prevalence that gave rise to the current lung cancer mortality (personal communication, 16/6/2005). *D*_*NS *_was then estimated as follows:

*D*_*NS *_= *D*_*P*_/((1 - *p*_*e *_+ *p*_e_*RR_S_)

and *D*_*S *_was estimated as for AMI and stroke.

The relative risk of lung cancer for smokers versus never smokers was estimated from data in the Appendix of Peto et al. [24]. For never-smokers, the smoothed never-smoker rates were used. It was assumed that the rates of death from lung cancer among smokers and never smokers measured in the American Cancer Society (ACS) Cancer Prevention Study (CPS-II) applied to the Australian population. The relative risk of COPD for smokers was obtained from English et al. [23] and was 9.8 for males and females. This is a conservative estimate compared with the estimates of 14.1 for males and 14.8 for females obtained by averaging the relative risks for ages ≥ 50 reported in Peto et al. [24].

#### Disease risks for quitters

##### Stroke and AMI

For stroke and AMI, the non-linear models published in Hurley [4] were used to estimate relative risks after smoking cessation. These non-linear models were updated versions of the models developed by Lightwood and Glantz for an analysis of the benefits of smoking cessation on stroke and AMI hospitalisation costs in the United States[25]. Hurley fitted non-linear models of the risks, RR(t) of AMI and stroke for ex-smokers relative to never smokers as a function of time, t, months since quitting. For the QBM, these functions were transformed to express risks for ex-smokers relative to current smokers, and are shown by year since quitting in Table 2. Note that the original data for fitting these models were available separately for males and females for AMI, but for stroke, only data for males and females combined were available.

**Table 2:** Risks of AMI and stroke in quitters relative to smokers by time since quitting*

**Year since quitting**	**Estimated RR**		
	**AMI**	**AMI**	**Stroke**

	**Males**	**Females**	**Males and Females**

1	0.66	0.66	0.75
2	0.49	0.49	0.63
3	0.41	0.41	0.57
4	0.37	0.36	0.54
5	0.35	0.34	0.53

* Source: Hurley[4]

##### Lung Cancer and COPD

Risk ratios for lung cancer for ex-smokers versus current smokers for specified time-periods since quitting were sourced from Peto et al. [26] and from Table F on the corresponding website. An exponential model to describe the decrease in risk of lung cancer over time was developed, and we assumed that this function also described the decrease in risk of COPD. We assumed that the relative risk, rr(t) of developing lung cancer for an ex-smoker who ceased smoking t months ago relative to a current smoker could be expressed as:

rr(t) = [(1- rr_∞_)]*e^-t/τ ^+ rr_∞_

where rr_∞ _is the relative risk of lung cancer for a never-smoker versus a current smoker, and τ is a slope parameter which is inversely proportional to the rate at which the relative risk decreases with time since quitting. Due to the paucity of data available, this model assumes that the risk for an ex-smoker eventually becomes the same as that of a never-smoker. The values of rr_∞ _were taken to be 0.03 and 0.05 respectively for males and females, as given in Peto et al. [26]. The values of τ were estimated by fitting the following non-linear model to the data.

ln(rr(t)) = ln([(1- rr_∞_)]*e^-t/τ ^+ rr_∞ _+ ε

The non-linear regression procedure (Levenberg-Marquardt estimation method) in the SPSS statistical software package was used. The natural logarithms of the relative risks were weighted proportionally to the inverse of their variances, which were estimated from the reported confidence intervals for the relative risks.

The estimated values of τ were 162 (95% CI: 129 – 195) for males, and 100 (95% CI: 61 – 139) for females. The reported and estimated relative risks are given in Table 3.

**Table 3:** Risk of lung cancer in quitters relative to smokers by time since quitting

**Group**	**Years since quitting**	Reported RR*	**Estimated RR**	**Difference**
Males	< 10	0.66	0.70	-0.04
	10 – 19	0.42	0.35	0.07
	20 – 29	0.18	0.18	0.00
	≥ 30	0.08	0.10	-0.02
Females	< 10	0.69	0.57	0.12
	10 – 19	0.21	0.21	0.00
	≥ 20	0.05	0.08	-0.03

* Source: Peto et al.[26]

### Mortality risks

#### Probabilities of death for people with smoking-related diseases

##### AMI

The probability of dying in hospital following the first AMI was calculated from the hospitalisation data sourced from the AIHW[16]. The probability of dying before reaching hospital was estimated by assuming that all first AMI deaths occurred before reaching hospital or in hospital, on the basis that a death post discharge would be due to a second or subsequent AMI or due to other causes.

The AMI case fatality rate, for estimation of the probability of dying in the first year after discharge from hospital and in subsequent years, was calculated in DisMod II. Inputs were the AMI incidence probabilities (described above under **Probabilities of smoking-related diseases**), the IHD-specific population mortality rates, [13] and the remission rate (which was assumed to be zero). It was assumed in males that 70% of case fatalities were due to the first AMI and in females that 80% were due to the first AMI. The AMI case fatality rates were adjusted accordingly to provide case fatality rates due to second or subsequent AMIs, and converted to probabilities using the formula in Section 3. These are the probabilities of someone who has had an AMI dying from an AMI, and were assumed to be the same for smokers and quitters. The probabilities of dying of causes other than AMI were then added to these probabilities. For quitters, the risk of death from other causes was assumed to decline over time relative to smokers, according to the function described below under ***Mortality risks (other causes)***.

##### Stroke

The probabilities of death following a stoke were based on outcomes for two cohorts of patients enrolled in the Perth Community Stroke Study, a population-based study in the state of Western Australia, in 1989–90 and 1995–96. These were the only Australian stroke survival data available. The probability of death in the first 30 days post stroke was 22.3% in the first cohort [27] and 22.5% in the second, [28] so an estimate of 22% was used in the QBM. The probabilities of death for the 1995–96 cohort were used for the first five years post-stroke and data for the 1989–90 cohort were used for the 6–10 year post-stroke period. The probabilities 10 years post-stroke were applied to all subsequent years. As these studies recorded deaths from all causes for a cohort of people (smokers, quitters and never-smokers) who had experienced a stroke, it was not necessary to add the probability of dying from other causes, and the same death probabilities were used for both smokers and quitters.

##### Lung cancer

The probabilities of death following a diagnosis of lung cancer were calculated from Victorian Cancer Registry survival data, for cases diagnosed between 1994 and 1999 (data supplied by Professor Dallas English, Cancer Council Victoria, 12/8/2005). Probabilities of death were calculated by taking a weighted average of the probabilities in each of the relevant years of follow-up, with weights proportional to the effective number at risk during each period. Probabilities were assumed to be constant after the first five years post-diagnosis, to overcome the instability in the data due to the small numbers of people being followed in these years. The probabilities of death 10 years post diagnosis were applied to all subsequent years. No data were available in the tenth year after diagnosis, for males and females over the age of 80, so the probabilities of death in the tenth year were estimated by the probabilities of death in the previous year (i.e. the weighted average of the probabilities for years 6 to 9 inclusive). The probabilities of death 10 years post diagnosis were applied to all subsequent years. The Cancer registry recorded deaths from all causes for a cohort of people (smokers, quitters and never-smokers) who had been diagnosed with lung cancer, so, as for the stroke death probabilities, it was not necessary to add the probability of dying from other causes, and the same death probabilities were used for both smokers and quitters.

##### COPD

COPD case fatality rates were estimated in DisMod II using the estimated COPD incidence for 2001 (see **Probabilities of smoking-related diseases**, above), COPD remission rates (assumed to be zero) and COPD-specific population mortality rates from the AIHW GRIM books [13] as input. The case fatality rates, which were assumed to be the same for smokers and quitters, were converted to probabilities and the probabilities of dying of causes other than COPD were then added to these probabilities. For quitters, the risk of death from other causes was assumed to decline over time relative to smokers, according to the function described below under ***Mortality risks (other causes)***.

#### Mortality risks (other causes)

The other mortality risk data needed to analyse the QBM are the transition probabilities from the state "well" to "dead" for smokers and quitters. We refer to these risks as "other causes" mortality, as the risk of dying from any of the four specified smoking-related diseases is excluded. We took the following approach to estimating these probabilities:

• Age-group, sex-specific mortality probabilities for all causes and for AMI (ICD 10 codes I20–I25), stroke (I60–I69), lung cancer (C33, C34) and COPD (J41–J44), for 2001 for the Australian population were sourced from the AIHW GRIM books [13].

• Smoker-specific mortality probabilities for the four smoking-related diseases were estimated using the methodology described under ***Disease risks for smokers***, assuming that the relative risks for mortality were the same as the relative risks for incidence of the specified diseases and substituting mortality probabilities for disease probabilities. To calculate all causes mortality risk for smokers, we used the same methodology, and sourced relative risks from the American Cancer Society (ACS) Cancer Prevention Study (CPS-II) data reported in Table 3 of Taylor et al. [29] Values for age less than 50 years were applied to age-groups 30–34 to 45–49 years. Relative risks were assumed to be one for age less than 30 years, as no participants in the CPS -II were younger than 30 years.

• The probability of a smoker dying of "other causes" (i.e. not AMI, stroke, lung cancer or COPD) was estimated by subtracting the four disease-specific mortality probabilities for smokers from the all causes mortality probability for smokers.

• Quitters' mortality risks for other causes were estimated by developing a function describing the decline in risk of death from all causes for quitters relative to smokers and applying the function to the probability of death from other causes for smokers. Data for the decline in all causes mortality risk for quitters relative to smokers were also sourced from the ACS CPS-II data reported by Taylor et al. [29]. Relative risks were given for five age-groups for males and females separately, and for four time-periods since quitting: 3–5 years, 6–10 years, 11–15 years and > 16 years. For the first three time periods the relative risk was taken to apply at the midpoints of the intervals, i.e. at 4, 8 and 13 years respectively. For the final interval, the relative risk was taken to apply at 20 years. As for lung cancer, it was assumed that the relative risk, rr(t) of dying for an ex-smoker who ceased smoking t months ago relative to a current smoker could be expressed as

rr(t) = (1- rr_∞_)*e^-t/τ ^+ rr_∞_

where rr_∞ _is the relative risk of death for a never-smoker versus a current smoker, and τ is a slope parameter which is inversely proportional to the rate at which the relative risk decreases with time since quitting. Again this model assumes that the risk of death for an ex-smoker eventually becomes the same as that of a never-smoker.

The relative risks and confidence intervals reported in Table 3 in Taylor et al. [29] were first expressed relative to never-smokers. Letting RR(t) represent the risk of death for an ex-smoker who ceased smoking t months ago relative to a never-smoker, then

RR(t) = rr(t) *RR_0_

where RR_0 _= RR(0), the relative risk of death for a current smoker versus a never-smoker. By definition, RR_0 _= 1/rr_∞_. Thus

RR(t) = (RR_0_-1)*e^-t/τ ^+ 1

The values of τ were estimated by fitting the non-linear model

ln(RR(t)) = ln((RR_0_-1)*e^-t/τ ^+ 1) + ε

to the data in Table 3 in Taylor et al. [29]. The parameters RR_0 _and τ were allowed to assume separate values for each sex and age-group, and the regression errors (ε) were assumed to be independent. Again, the regression analyses were carried out using the non-linear regression procedure in the SPSS statistical software package, using the Levenberg-Marquardt estimation method. The natural logarithms of the relative risks were weighted proportional to the inverse of their variances. These variances were estimated from the reported confidence intervals for the relative risks.

For each sex and age-group, the values of rr_∞ _were obtained directly by taking the inverse of the risks for current smokers relative to never-smokers reported in Taylor et al. These values of rr_∞ _could have been estimated by inverting the estimated parameters RR_0 _from the model-fitting procedure, however it was considered that the directly reported values would be more accurate that the estimated values. In reality, the estimated and reported values differed by < 3% in all cases.

The estimated values of τ and rr_∞ _are shown in Table 4 and the corresponding estimated relative risks for each sex and age-group are compared in Table 5 with the values obtained from Table 3 in Taylor et al. [29] by dividing the reported risks (RR) for quitters relative to never-smokers by the reported risks for current smokers relative to never-smokers.

**Table 4:** Model parameters* for function describing the decline in all causes mortality after quitting

**Sex**	**Age**	**Estimated τ**	**Asymptotic Standard Error**	**Asymptotic 95% CI**	**rr**_∞_
Male	< 50	63	60	-60 – 186	0.427
	50 – 59	110	22	64 – 156	0.355
	60 – 69	150	16	117 – 184	0.357
	70 – 79	187	22	143 – 231	0.397
	≥ 80	202	57	86 – 318	0.553
Female	< 50	101	143	-191 – 393	0.595
	50 – 59	71	31	7 – 134	0.431
	60 – 69	132	22	88 – 176	0.398
	70 – 79	146	21	103 – 190	0.407
	≥ 80	190	52	84 – 296	0.553

* The model fitted was: ln(RR(t)) = ln((RR_0_-1)*e^-t/ τ^+ 1) + ε, where RR(t) = the risk of death for an ex-smoker who ceased smoking t months ago relative to a never-smoker; RR_0_= RR(0), the relative risk of death for a current smoker versus a never-smoker. rr_∞ _= the relative risk of death for a never-smoker versus a current smoker (By definition, RR_0 _= 1/rr_∞_). τ is a slope parameter which is inversely proportional to the rate at which the relative risk decreases with time since quitting.

**Table 5:** Risk of all causes mortality in quitters relative to smokers by time since quitting

**Sex**	**Age**	**Years since quitting**	Reported rr*	**Estimated rr**	**Difference**
Male	< 50	3 – 5	0.55	0.67	-0.12
		6 – 10	0.62	0.54	0.08
		11 – 15	0.40	0.47	-0.07
		≥ 16	0.41	0.44	-0.03
	50 – 59	3 – 5	0.68	0.75	-0.07
		6 – 10	0.80	0.61	0.19
		11 – 15	0.53	0.50	0.03
		≥ 16	0.40	0.42	-0.02
	60 – 69	3 – 5	0.76	0.81	-0.05
		6 – 10	0.78	0.68	0.10
		11 – 15	0.63	0.58	0.05
		≥ 16	0.44	0.48	-0.04
	70 – 79	3 – 5	0.79	0.85	-0.06
		6 – 10	0.83	0.75	0.08
		11 – 15	0.76	0.65	0.11
		≥ 16	0.52	0.56	-0.04
	≥ 80	3 – 5	0.62	0.90	-0.28
		6 – 10	0.86	0.82	0.04
		11 – 15	0.88	0.75	0.13
		≥ 16	0.66	0.68	-0.02
Female	< 50	3 – 5	0.92	0.83	0.09
		6 – 10	0.65	0.74	-0.09
		11 – 15	0.66	0.68	-0.02
		≥ 16	0.67	0.63	0.04
	50 – 59	3 – 5	0.76	0.70	0.06
		6 – 10	0.56	0.57	-0.01
		11 – 15	0.53	0.49	0.04
		≥ 16	0.41	0.45	-0.04
	60 – 69	3 – 5	0.82	0.80	0.02
		6 – 10	0.75	0.68	0.07
		11 – 15	0.63	0.57	0.06
		≥ 16	0.44	0.49	-0.05
	70 – 79	3 – 5	0.63	0.82	-0.19
		6 – 10	0.73	0.70	0.03
		11 – 15	0.69	0.60	0.09
		≥ 16	0.49	0.52	-0.03
	≥ 80	3 – 5	0.91	0.89	0.02
		6 – 10	0.85	0.81	0.04
		11 – 15	0.81	0.74	0.07
		≥ 16	0.67	0.68	-0.01

* Source: Taylor et al.[29]: rr obtained by dividing the reported risks (RR) for quitters relative to never-smokers by the reported risks for current smokers relative to never-smokers in each sex and age-group.

#### Health care costs for smoking-related diseases

The health care cost estimates used in the QBM for the four specified smoking-related diseases are summarised in Table 6, and the sources of these estimates are described below. All costs in the QBM are in Australian dollars, A$. We conducted searches of the Medline database and Internet to identify Australian studies of the cost per patient for the four diseases. For AMI and COPD, no suitable Australian data could be found. Therefore, for AMI, we derived costs from Australian Refined Diagnosis Related Group (AR-DRG) and health service utilisation data. For COPD, we used a Canadian estimate.

**Table 6:** Health care cost estimates

	Costs*	
**Disease state**	**Males**	**Females**

**AMI**		
Hospital admission	$10,200	$8,800
Remainder of Year 1	$3,400	$2,700
Year 2 and later	$1,400	$1,400
**Stroke**		
First 30 days	$5,900	
Remainder of Year 1	$11,600	
Year 2 and later	$4,500	
**Lung cancer**		
On diagnosis (transition costs to lung ca)	$23,400	
Year 1 and later	$5,000	
Terminal care (transition cost to death)	$7,000	
**COPD**		
Annual cost	$2,200	

* 2001 Australian dollars

##### AMI

Hospitalisation costs were used as estimators of the AMI-related health care costs for the first year. The AR-DRG cost weights for the actual AR-DRGs for hospital admissions in 2001–2 for 35–64 year olds were supplied by the AIHW for the previous study of AMI and stroke hospitalisation cost savings associated with tobacco control by Hurley [4]. AR-DRG cost weights were available for admissions with a primary diagnosis of AMI, with or without revascularisation through percutaneous coronary intervention (PCI) or coronary artery bypass grafting (CABG). The AIHW report on coronary heart disease in Australia by Mathur [30] includes data on the proportion of 40–84 year old patients in Perth in 1997 who received either of these coronary revascularisation procedures at various time points after their AMI (i.e. during their acute admission, or within 30 days, 90 days and 1 year of the acute admission). These proportions and the relevant cost weights were combined to estimate costs for "Hospital admission" and "Remainder of year 1", separately for men and women.

Costs for "Year 2 and later" were assumed to be ambulatory care costs only. It was assumed that a patient would be prescribed one of the statin drugs plus one other drug and would visit a general practitioner twice a year. Based on average unit costs for these pharmaceuticals and services, the annual cost was estimated to be A$1,400.

These estimates for AMI are conservative, as hospitalisation costs after the first year are not included. Many patients who survive their first AMI subsequently die from ischaemic heart disease, and in some instances would be hospitalised before death.

##### Stroke

Costs were based on NEMESIS, the prospective Australian study used as a source of stroke incidence probabilities [17]. Costs from NEMESIS were adjusted to 2001 dollars on the basis of the health index of the Consumer Price Index (CPI) for Australia [31].

Dewey et al. reported a range of cost estimates derived from NEMESIS, including first-year costs for first-ever-in-a-lifetime stroke, and lifetime costs. For the QBM, the direct health care cost in the first 30 days after a stroke was estimated as the product of the proportion of incident cases admitted to hospital (88%, From Table 1 in Dewey et al. [32]) and mean patient-specific acute hospitalisation costs (A$6,651 from Table 1 in Dewey et al. [32]), giving an estimate of A$5,900 after CPI adjustment. The cost for the remainder of Year 1 was estimated by subtracting indirect costs and acute hospitalisation costs from the first-year cost for a first-ever stroke (A$18,956), giving an estimate of A$11,600 after CPI adjustment. The cost for subsequent years was estimated by dividing lifetime direct health care costs of stroke (A$41,762) by the average survival time post stroke (6.4 years assuming 79% risk of death at 10 years and an exponential distribution for survival), and subtracting first-year costs. After price adjustment this gave an estimate of A$4,500 per year for Year 2 and later.

##### Lung Cancer

Costs for diagnosis and first-line therapy were taken from an Australian study by Rosenthal et al. [33] and adjusted to 2001 prices from 1990 prices using the health index of the CPI (as for stroke). Total lifetime costs were estimated assuming that these initial costs represented the same proportion of total costs (66.2%) as in a more recent UK study by Oliver et al. [34]. Annual costs and terminal costs were then estimated using the proportions from the Oliver study.

##### COPD

Annual costs were estimated from the Canadian results for the Confronting COPD study[35]. This study surveyed over 400 Canadian patients with COPD and ascertained their health service utilisation over the 12-month period prior to the survey. Direct health care costs were converted to Australian dollars on the basis of Purchasing Power Parities for 2001 [36].

### Utilities for smoking-related diseases

For stroke, a pooled estimate from a meta analysis was used (see below) For AMI, lung cancer and COPD, the means of the utility scores reported in the catalogues of preferences published by the CEA Registry [37] and more recent publications were used as estimates of the utility of health states in the QBM associated with the disease. The CEA Registry, now operated by the Institute for Clinical Research and Health Policy Studies at the Tufts-New England Medical Center, is recognised as a standard source for disease utility estimates [37]. This group has conducted exhaustive literature searches for published cost-utility studies, and have published two on-line catalogues of the preference scores (utilities) from these studies. Phase I covers literature published between 1976–1997, and Phase II covers the period 1998–2001. We sourced preference scores from these catalogues that came from studies where preferences had been measured. Studies where the author or clinicians had estimated utilities were not included. Medline searches were conducted for the period from 2002 to October 2005 to identify any more recent studies reporting preference scores for these diseases that would not have been included in the Phase 11 catalogue.

#### AMI

A utility of 0.84 was assigned to all AMI states. The mean of 0.84 came from seven studies: five from the 1976–1997 Catalogue (range of values: 0.87–0.93) and two from the 1998–2001 Catalogue (range: 0.58–0.88).

#### Stroke

All stroke health states were assumed to have a utility of 0.45. This estimate was sourced from the meta-regression of 20 articles (contributing 53 unique quality of life weights) by Tengs and Lin [38]. The value of 0.45 was for moderate stroke when the scale bounds were death to normal health. It is very similar to the estimate of 0.47 for mean AQoL (Assessment of Quality of Life) score in the Australian NEMESIS study for 266 incident cases at 2 years post stroke [39].

#### Lung Cancer

A utility of 0.63 was assumed for lung cancer. This was the mean of four estimates. One came from the 1976–1997 Harvard Catalogue of preference scores (value: 0.73), one came from the 1998–2001 Catalogue (value:0.58), and two were published more recently (range: 0.58–0.62)[40,41].

#### COPD

A utility of 0.61 was assumed for COPD. This was the mean of estimates from three studies, all in the 1998–2001 Catalogue of preference scores (range: 0.38–0.74).

### Sensitivity analyses

One-way sensitivity analyses were conducted by varying, by ± 10%, the values of each of the 20 key parameters used to calculate QALYs and total health care costs. A multi-way sensitivity analysis was also conducted in which AMI and stroke incidences were decreased by 10%, to reflect population trends, and AMI and stroke survival, costs and utilities were increased by 10% to reflect the availability of more costly treatments, especially for AMI, that improve survival and quality of life.

## Results

The predicted life-years, QALYs and health care costs for males and females in each five year age-group between ages 15 and 74, who are followed for 10 years are presented in Tables 7, 8 and 9, respectively. A full set of results in EXCEL spreadsheet format is also available from the authors on request. Results are presented with no discounting, and 3% and 5% per annum discount rates. The data for the age-groups beyond age 74 were not included in the summary in order to give a full ten-year follow-up for all age-groups. The outcomes for smokers, quitters and the difference between the two are presented. For example, if a male aged between 50 and 54 quits smoking, the QBM predicts that in the following 10 years he will gain 0.1 life-years and 0.1 QALYs, and the health care cost saving associated with his reduced risk of AMI, stroke, lung cancer and COPD would be $861, the difference between $2,477 if he had continued to smoke and $1,616 having become a quitter (5% per annum discounting). Cost savings in the 10 years after quitting increase with the age at quitting, as older ages are associated with higher risks of developing the diseases under study. However, this pattern is not seen with all of the disease-specific health care costs because the QBM is a competing risks model, and therefore a quitter, who, through quitting, is less likely to get one of the four diseases, may be more likely to survive long enough to be at risk of developing the most expensive of the four diseases.

**Table 7:** Predicted life-years for males and females after 10 years follow-up

**Males**									
	**No discounting**			**3% pa discounting**			**5% pa discounting**		

**Age-group**	**Smoker**	**Quitter**	**Difference**	**Smoker**	**Quitter**	**Difference**	**Smoker**	**Quitter**	**Difference**

15–19	10.0	10.0	0.0	8.6	8.6	0.0	7.9	7.9	0.0
20–24	9.9	10.0	0.0	8.6	8.6	0.0	7.9	7.9	0.0
25–29	9.9	9.9	0.0	8.6	8.6	0.0	7.9	7.9	0.0
30–34	9.9	9.9	0.0	8.6	8.6	0.0	7.8	7.9	0.0
35–39	9.9	9.9	0.0	8.5	8.6	0.0	7.8	7.8	0.0
40–44	9.8	9.9	0.1	8.5	8.5	0.1	7.8	7.8	0.0
45–49	9.7	9.8	0.1	8.4	8.5	0.1	7.7	7.8	0.1
50–54	9.5	9.6	0.1	8.3	8.4	0.1	7.6	7.7	0.1
55–59	9.2	9.4	0.2	8.0	8.2	0.2	7.3	7.5	0.2
60–64	8.7	9.0	0.3	7.6	7.9	0.2	7.0	7.2	0.2
65–69	8.1	8.5	0.4	7.1	7.4	0.3	6.6	6.8	0.3
70–74	7.3	7.8	0.5	6.4	6.8	0.4	6.0	6.3	0.3

**Females**									

	**No discounting**			**3% pa discounting**			**5% pa discounting**		

**Age-group**	**Smoker**	**Quitter**	**Difference**	**Smoker**	**Quitter**	**Difference**	**Smoker**	**Quitter**	**Difference**

15–19	10.0	10.0	0.0	8.6	8.6	0.0	7.9	7.9	0.0
20–24	10.0	10.0	0.0	8.6	8.6	0.0	7.9	7.9	0.0
25–29	10.0	10.0	0.0	8.6	8.6	0.0	7.9	7.9	0.0
30–34	9.9	10.0	0.0	8.6	8.6	0.0	7.9	7.9	0.0
35–39	9.9	9.9	0.0	8.6	8.6	0.0	7.9	7.9	0.0
40–44	9.9	9.9	0.0	8.6	8.6	0.0	7.8	7.9	0.0
45–49	9.8	9.9	0.0	8.5	8.5	0.0	7.8	7.8	0.0
50–54	9.7	9.8	0.1	8.4	8.5	0.1	7.7	7.8	0.1
55–59	9.5	9.7	0.1	8.3	8.4	0.1	7.6	7.7	0.1
60–64	9.2	9.4	0.2	8.0	8.2	0.2	7.4	7.5	0.1
65–69	8.7	9.0	0.3	7.6	7.9	0.2	7.0	7.2	0.2
70–74	8.0	8.4	0.4	7.0	7.4	0.3	6.5	6.8	0.3

**Table 8:** Predicted QALYs for males and females after 10 years follow-up

**Males**									
	**No discounting**			**3% pa discounting**			**5% pa discounting**		

**Age-group**	**Smoker**	**Quitter**	**Difference**	**Smoker**	**Quitter**	**Difference**	**Smoker**	**Quitter**	**Difference**

15–19	9.9	9.9	0.0	8.6	8.6	0.0	7.9	7.9	0.0
20–24	9.8	9.9	0.0	8.5	8.6	0.0	7.8	7.8	0.0
25–29	9.8	9.8	0.0	8.5	8.5	0.0	7.8	7.8	0.0
30–34	9.8	9.8	0.1	8.5	8.5	0.0	7.7	7.8	0.0
35–39	9.7	9.8	0.1	8.4	8.5	0.1	7.7	7.8	0.0
40–44	9.7	9.8	0.1	8.4	8.5	0.1	7.7	7.8	0.1
45–49	9.5	9.7	0.1	8.3	8.4	0.1	7.6	7.7	0.1
50–54	9.3	9.5	0.2	8.1	8.2	0.2	7.4	7.5	0.1
55–59	8.9	9.2	0.3	7.7	8.0	0.2	7.1	7.3	0.2
60–64	8.4	8.8	0.4	7.3	7.6	0.3	6.7	7.0	0.3
65–69	7.7	8.2	0.5	6.8	7.2	0.4	6.3	6.6	0.3
70–74	6.8	7.4	0.6	6.1	6.5	0.5	5.6	6.0	0.4

**Females**									

	**No discounting**			**3% pa discounting**			**5% pa discounting**		

**Age-group**	**Smoker**	**Quitter**	**Difference**	**Smoker**	**Quitter**	**Difference**	**Smoker**	**Quitter**	**Difference**

15–19	10.0	10.0	0.0	8.6	8.6	0.0	7.9	7.9	0.0
20–24	9.9	10.0	0.0	8.6	8.6	0.0	7.9	7.9	0.0
25–29	9.9	9.9	0.0	8.5	8.6	0.0	7.8	7.8	0.0
30–34	9.8	9.9	0.1	8.5	8.5	0.0	7.8	7.8	0.0
35–39	9.8	9.9	0.0	8.5	8.5	0.0	7.8	7.8	0.0
40–44	9.8	9.9	0.1	8.5	8.5	0.0	7.8	7.8	0.0
45–49	9.7	9.8	0.1	8.4	8.5	0.1	7.7	7.7	0.1
50–54	9.5	9.7	0.1	8.3	8.4	0.1	7.6	7.7	0.1
55–59	9.3	9.5	0.2	8.1	8.3	0.2	7.4	7.6	0.1
60–64	9.0	9.2	0.3	7.8	8.0	0.2	7.2	7.4	0.2
65–69	8.4	8.8	0.4	7.4	7.7	0.3	6.8	7.0	0.3
70–74	7.6	8.2	0.5	6.7	7.2	0.4	6.2	6.6	0.4

**Table 9:** Predicted health care costs* for males and females after 10 years follow-up.

**Males**									
	**No discounting**			**3% pa discounting**			**5% pa discounting**		

**Age-group**	**Smoker**	**Quitter**	**Difference**	**Smoker**	**Quitter**	**Difference**	**Smoker**	**Quitter**	**Difference**

15–19	$268	$178	$90	$215	$144	$72	$187	$125	$62
20–24	647	$487	$160	$529	$400	$128	$465	$353	$112
25–29	$811	$635	$176	$668	$527	$142	$591	$468	$123
30–34	$905	$650	$255	$742	$536	$206	$654	$474	$179
35–39	$1,055	$730	$325	$873	$609	$264	$775	$543	$231
40–44	$1,353	$823	$530	$1,112	$682	$430	$982	$606	$376
45–49	$2,211	$1,378	$833	$1,826	$1,147	$679	$1,618	$1,022	$596
50–54	$3,355	$2,160	$1,195	$2,786	$1,808	$978	$2,477	$1,616	$861
55–59	$4,457	$2,984	$1,474	$3,731	$2,516	$1,215	$3,335	$2,260	$1,075
60–64	$5,384	$3,715	$1,669	$4,532	$3,150	$1,382	$4,065	$2,840	$1,226
65–69	$6,363	$4,514	$1,848	$5,377	$3,842	$1,535	$4,836	$3,472	$1,364
70–74	$7,595	$5,530	$2,066	$6,469	$4,734	$1,735	$5,846	$4,293	$1,553

**Females**									

	**No discounting**			**3% pa discounting**			**5% pa discounting**		

**Age-group**	**Smoker**	**Quitter**	**Difference**	**Smoker**	**Quitter**	**Difference**	**Smoker**	**Quitter**	**Difference**

15–19	$73	$38	$35	$57	$30	$27	$49	$26	$23
20–24	$333	$192	$141	$269	$156	$113	$234	$137	$98
25–29	$681	$435	$246	$560	$361	$199	$494	$320	$174
30–34	$890	$606	$284	$736	$505	$231	$652	$450	$202
35–39	$826	$580	$247	$685	$485	$200	$608	$434	$175
40–44	$958	$564	$393	$784	$467	$317	$690	$413	$276
45–49	$1,510	$932	$578	$1,249	$780	$469	$1,107	$697	$410
50–54	$2,080	$1,264	$815	$1,724	$1,060	$664	$1,531	$949	$582
55–59	$2,739	$1,669	$1,070	$2,284	$1,409	$875	$2,036	$1,266	$770
60–64	$3,562	$2,193	$1,369	$2,979	$1,854	$1,125	$2,660	$1,667	$993
65–69	$4,342	$2,769	$1,573	$3,649	$2,352	$1,296	$3,270	$2,123	$1,147
70–74	$5,429	$3,489	$1,940	$4,565	$2,956	$1,609	$4,091	$2,662	$1,429

* 2001 Australian dollars

In Table 10, all outcomes predicted by the QBM for a 50–54 year old male followed for 10 years are presented. As the cumulative probabilities of disease and death cannot be discounted, none of the results in this Table have been discounted. The Table shows that when a man quits smoking at this age, the cost savings associated with avoiding an AMI or stroke are substantially higher than the cost savings associated with avoiding COPD or lung cancer. By quitting, the hypothetical man's probability of being diagnosed with  one of the four smoking-associated diseases in the following 10 years is  reduced by 40%, and his probability of dying is reduced by 35%.

**Table 10:** Predicted outcomes for a male aged between 50–54 years after 10 years follow-up.*

**Outcome**	**Smoker**	**Quitter**	**Difference**
Life-years	9.5	9.6	0.1
QALYs	9.3	9.5	0.2
**Health care costs^† ^avoided**			
AMI costs	$783	$340	$443
COPD costs	$875	$706	$169
Lung Ca costs	$861	$617	$244
Stroke costs	$837	$498	$339
**Total costs**	**$3,355**	**$2,160**	**$1,195**
**Cumulative probability of disease**			
AMI	0.058	0.024	0.034
COPD	0.089	0.065	0.025
Lung Ca	0.025	0.016	0.009
Stroke	0.037	0.021	0.015
**Any of the above 4 diseases**	**0.209**	**0.126**	**0.083**
**Cumulative probability of death**			
AMI	0.017	0.006	0.011
COPD	0.006	0.004	0.002
Lung Ca	0.016	0.011	0.005
Stroke	0.017	0.010	0.007
**Any of the above 4 diseases**	**0.057**	**0.031**	**0.026**
Causes other than the above	0.058	0.044	0.014
**Total deaths**	**0.115**	**0.075**	**0.04**

* No discounting

† In 2001 Australian dollars

Table 11 summarises the outcomes for 1,000 quitters chosen at random from a population of smokers aged between 15 and 74 years at the time of quitting and followed for 10 years. The reference population (see Table 12), was the estimated Australian population of smokers in 2001 aged between 15 and 74 years of age, obtained by multiplying the population estimates for males and females separately by the estimated prevalence of smoking in the five-year age-groups. Overall there were an estimated 2.1 million male smokers and 1.6 million female smokers in this age range. Their age distribution is given in Table 11, and, as expected, is more heavily skewed towards the younger ages, because of the greater population density and increased prevalence of smoking at the younger ages. For males, smoking prevalence rates ranged from 31% for 15–19 year olds to 12% for 70–74 year olds, and correspondingly from 23% to 11% in females. These outcomes can be conceptualised as standardised measures of the effectiveness of a quitting program.

**Table 11:** Outcomes predicted by the QBM for 1,000 quitters*

**Category of benefit**	**Per 1,000 Males**	**Per 1,000 Females**	**Per 1,000 Individuals**
Life-years saved	57	35	47
QALYs saved	85	62	75
**Health care costs^† ^avoided**			
AMI costs	$131 k	$48 k	$95 k
COPD costs	$86 k	$102 k	$93 k
Lung Ca costs	$71 k	$71 k	$71 k
Stroke costs	$119 k	$107 k	$114 k
**Total costs**	**$408 k**	**$328 k**	**$373 k**
**Cases of disease avoided**			
AMI	14	6	11
COPD	18	20	19
Lung Ca	3	3	3
Stroke	8	7	8
**Any of the above 4 diseases^&^**	**43**	**37**	**40**
**Deaths avoided**			
AMI^	6	2	4
COPD^	2	1	1
Lung Ca^	2	2	2
Stroke^	3	4	3
**Any of the above 4 diseases^**	**12**	**9**	**11**
Causes other than the above	9	5	7
**Total deaths**	**21**	**14**	**18**

* Outcomes predicted by the QBM for 1,000 quitters chosen at random from the population of smokers aged between 15 and 74 years at the time of quitting and followed for 10 years. Life-years, QALYs and costs discounted at 5% per annum; cases avoided and deaths not discounted.

† In 2001 Australian dollars

^&^Cases and deaths have been rounded to the nearest integer and hence the total presented may not equal the sum of the components

^ These are deaths for people with these specific diseases

**Table 12:** Age-distribution of reference population of smokers

Age-group	Males	Females	Individuals
15–19	10.2%	9.4%	9.9%
20–24	11.9%	11.5%	11.7%
25–29	12.6%	12.7%	12.6%
30–34	12.6%	12.7%	12.6%
35–39	12.6%	11.8%	12.2%
40–44	10.6%	12.7%	11.5%
45–49	8.5%	8.3%	8.4%
50–54	7.5%	8.1%	7.8%
55–59	5.9%	4.8%	5.4%
60–64	3.8%	4.1%	3.9%
65–69	2.1%	1.7%	1.9%
70–74	1.8%	2.3%	2.0%

Overall, for every 1,000 males chosen at random from the reference population who quit smoking, there is a an average saving of A$408,000 in health care costs associated with AMI, COPD, lung cancer and stroke, and a corresponding saving of A$328,000 for every 1,000 female quitters. This translates to an average saving of A$373,000 per 1,000 random quitters. Overall 40 of these individuals will be spared a diagnosis of AMI, COPD, lung cancer or stroke in the first ten years following quitting, with an estimated saving of 47 life-years and 75 QALYs.

The results of the one-way sensitivity analyses are presented in Table 13. Increasing or decreasing any of the model parameters by 10% resulted in only small changes in the outcomes assessed in the sensitivity analyses. The predicted QALYs were most sensitive to a change in the utility of COPD, but a 10% decrease in the COPD utility estimate still only produced a 3.4% decrease in QALYs saved per 1000 random quitters over a 10 year follow-up period. Costs were most sensitive to a change in the rate at which a quitter's risk of lung cancer or COPD returned to that of a smoker. A 10% increase in τ, which corresponds to a less rapid reduction in the risk of these smoking associated diseases, was predicted to produce a 3.4% reduction in cost savings, and a 10% decrease in τ was predicted to produce a 4% increase in cost savings. The QBM remained robust when a multi-way sensitivity analysis was performed. The predicted QALY gain for 1000 random quitters decreased by only 7.9% and the predicted costs increased by only 2.4% when AMI and stroke risks were both decreased by 10%, and AMI and stroke survival probabilities, costs and utilities were all increased by 10%.

**Table 13:** One-way sensitivity analyses for QALYs and total health care costs

**Parameter changed**	**% Change in outcome given a 10% increase in relevant parameter**		**% Change in outcome given a 10% decrease in relevant parameter**	
	**QALYs**	**Costs**	**QALYs**	**Costs**

τ for AMI and Stroke	-1.0%	-1.1%	1.0%	1.1%
τ for lung cancer and COPD	-2.4%	-3.4%	2.8%	4.0%
τ for other cause mortality	-1.9%	0.1%	2.2%	-0.1%
Incidence AMI	1.7%	2.1%	-1.7%	-2.2%
Incidence Stroke	1.9%	2.8%	-1.9%	-2.8%
Incidence Lung Cancer	0.6%	1.8%	-0.6%	-1.8%
Incidence COPD	2.1%	2.0%	-2.1%	-2.0%
Mortality AMI	1.1%	-1.1%	-1.2%	1.1%
Mortality Stroke	0.4%	-1.2%	-0.5%	1.2%
Mortality Lung Cancer	0.2%	-0.2%	-0.2%	0.2%
Mortality COPD	0.2%	-0.2%	-0.2%	0.2%
Mortality Other Causes	2.3%	-0.7%	-2.3%	0.7%
Costs AMI		2.5%		-2.5%
Costs Stroke		3.1%		-3.1%
Costs Lung Cancer		1.9%		-1.9%
Costs COPD		2.5%		-2.5%
Utility AMI	-2.2%		2.2%	
Utility Stroke	-0.8%		0.8%	
Utility Lung Cancer	-0.2%		0.2%	
Utility COPD	-3.4%		3.4%	

## Discussion

The QBM predicts that the benefits of quitting smoking are substantial in the subsequent 10-year period. For every 1000 quitters, randomly selected from the Australian smoker population, savings of almost A$400,000 in health care costs, and prevention of 40 cases of the four most common smoking-associated diseases and 18 deaths can be expected. Sensitivity analyses indicated that these predictions are robust to plausible variations in the model parameters.

The validity of a model such as the QBM is dependent on, first, how well the model structure represents the outcomes of smoking and quitting in real life, and, second, on the accuracy of the model parameters. In developing the QBM, when assumptions about model structure or choices about data sources were needed, we took the most conservative course, i.e. we chose to under-estimate the adverse health and health care cost consequences of smoking and to under-estimate the benefits of quitting. In relation to disease incidence, health care costs and quality of life, the QBM considers only four of the hundreds of smoking-related diseases, [42] making the model conservative with respect to cost savings and QALYs, although these four diseases do account for over 80% of morbidity (and mortality) associated with smoking. Furthermore we only modelled the incidences and costs associated with the first of these four smoking-related diseases which occurred in an individual [12]. This is conservative, as people who have had an AMI, for example, might also subsequently have a stroke, COPD or lung cancer. However the mortality rates following a diagnosis of one of the four diseases did include deaths from any other causes.

The QBM only considers the impact of quitting on the smoker. This is also conservative, because smoking adversely affects others, in particular by increasing the risk of coronary heart disease in those exposed to environmental tobacco smoke [43] and increasing the risk of a pregnant woman who smokes having a low-birth-weight infant. Quitting smoking can reduce these risks, [44,45] but this was not incorporated in the QBM.

The most important parameters in the QBM are the exponential models describing the decline after quitting in the risk of the four smoking-associated diseases and *other causes *mortality. These models were all based on relative risks from studies involving very large numbers of subjects. For AMI and stroke, the models were previously developed by one of us (SH) [4] and were based on relative risks from all available published studies that met specified criteria. Relative risks for AMI were sourced from four case-control studies with more than 8000 cases and more than 16,000 controls, [46-49] and one cohort study involving almost 1000 cases and over 120,000 subjects [50]. For stroke, relative risks came from two cohort studies with over 600 cases and more than 120,000 subjects[51,52]. For lung cancer and COPD, the model was based on relative risks from a lung cancer case-control study with more than 600 cases and more than 2000 controls [26]. The mortality model was based on the ACS CPS II study of 1.2 million people [29].

The model structure and data sources in relation to mortality are also important aspects of the QBM. Mortality rates for the four smoking-associated diseases, were based on case fatality data from a variety of sources. To estimate the *other causes *mortality rates, cause-specific Australian population mortality rates for these four conditions were subtracted from the Australian *all causes *mortality rates. To estimate the *other causes *mortality rates for quitters we assumed that the decline in risk for quitters relative to smokers could be estimated by the function estimating the decline in risk for *all causes *mortality. If this decline in risk has been overestimated, we would have underestimated the number of quitters who would die of *other causes *and hence **overestimated **the benefit of quitting with respect to *other causes *mortality. However we would have correspondingly **underestimated **the benefit of quitting with respect to the incidence of the four smoking-related diseases and their health care costs, as the quitters would be at greater risk of these diseases through their reduced risk of death from *other causes*. This highlights the "competing risk" aspect of the model.

The QBM parameter estimates for disease probability, case fatality, disease cost and disease utility parameter estimates were based on the best available data. With the exception of COPD, the disease probability and case fatality data came either from Australian population-based studies or routine data collections, and the parameter estimates should therefore be reasonably accurate. The COPD incidence and fatality data were derived from Australian data using modelling and are therefore inherently less reliable. However, better estimates are unlikely to be available without a large scale prospective study because of the typically insidious onset of COPD.

Of the health care cost estimates, the stroke data came from the most reliable source, a comprehensive, prospective, population-based study[32]. The AMI and lung cancer health care cost estimates were both based on Australian hospitalisation data, and are therefore under-estimates. There were no Australian data on the costs of managing patients with COPD, so data from the Canadian arm of an international study of COPD were used,[35] as Canada has a similar health care system to Australia. The disease utility estimates for lung cancer, stroke and AMI came from an international database and the stroke utility estimate came from a meta analysis.

So, in summary, the QBM under-estimates the benefits of quitting, as the model only considers the impact of quitting on a sub-set of the adverse health effects of smoking and the assumptions underpinning the parameter estimates were mostly conservative.

In this paper we have used the QBM to produce summaries of the benefits of quitting for an individual and for a randomly selected cohort of 1000 quitters. The model is also a tool that can be easily used to evaluate the consequences and cost-effectiveness of quitting programs. To estimate the impact of a quitting program on a particular outcome, such as QALYs, the number of quitters for each age-group and sex category is simply multiplied by the predicted difference between smokers and quitters for that outcome, and the products are summed over all age-sex categories. The QBM can also easily be adapted to incorporate different epidemiologic data. Key input tables for the QBM can be stored in a set of linked EXCEL spreadsheets, and imported into the TreeAge model to form a package. These input tables can be readily updated from the base year of 2001, for example, when more recent data becomes available. Similarly, a set of incidence, mortality and cost data for a different population could be input to obtain predictions of the benefits of quitting for that population.

The QBM also has a variety of other potential applications. For example, one of the major current challenges for tobacco control is the differential in smoking rates associated with socio-economic status. In Australia, for example, people in the occupation category regarded as having the lowest socioeconomic status are almost three time more likely to smoke than those in the highest socioeconomic occupation category[1]. The QBM could be used to compare the cost-effectiveness of tobacco control programs targeted at smokers of low socio-economic status with population-based programs.

## Conclusion

There were sufficient good quality data available for us to develop a comprehensive model of the health and health economic consequences of quitting smoking for Australians. The QBM can answer many of the questions posed by Australian policy-makers and health-care funders and will be a useful tool to evaluate tobacco control programs.

## Competing interests

The author(s) declare that they have no competing interests.

## Authors' contributions

Both SH and JM contributed to the design of the model. SH sourced the parameter estimates and JM wrote the TreeAge code and ran the model. SH drafted, and JM edited, the paper. Both authors approved the final version of the manuscript.
